# Investigating selective media for optimal isolation of *Brucella* spp. in South Africa

**DOI:** 10.4102/ojvr.v87i1.1792

**Published:** 2020-03-04

**Authors:** Maphuti B. Ledwaba, Okechukwu C. Ndumnego, Itumeleng Matle, Awoke K. Gelaw, Henriette van Heerden

**Affiliations:** 1 Department of Veterinary Tropical Diseases, Faculty of Veterinary Science, University of Pretoria, Pretoria, South Africa; 2 Africa Health Research Institute, Durban, South Africa; 3 Department of Bacteriology, Agricultural Research Council – Onderstepoort Veterinary Research Institute, Onderstepoort, South Africa

**Keywords:** Bovine brucellosis, selective media, Bacterial isolation

## Abstract

Bovine brucellosis in South Africa is caused mainly by *Brucella abortus biovar* (*bv*.) 1 and less frequently by *B. abortus bv.* 2. Bacterial isolation is regarded as the gold standard for diagnosis of *Brucella* species; however, it is not very sensitive. The aim of this study was to determine the selective medium with optimum antibiotic composition that will allow the growth of *Brucella* species (spp.) while inhibiting moulds, yeast and most, if not all, Gram-negative contaminants in South Africa. In the controlled experiment, modified Agrifood Research and Technology Center of Aragon (CITA) medium (mCITA) seemed to be the optimum selective medium for isolation of *Brucella* spp. as compared with Farrell’s medium (FM) and modified Thayer Martin (mTM), while FM inhibited the growth of most fungal and bacterial contaminants. Mean comparison between the three media used to culture *B. abortus* resulted in lower mean difference ranging from 0 to 2.33. In case of *Brucella ovis*, high mean difference was obtained when comparing FM with mCITA (10.33) and mTM (12). However, the mean differences of 0.67 and 1.67 were obtained when comparing mCITA and mTM media used to, respectively, culture pasteurised and raw milk spiked with *B. ovis*. Further optimisation at the Agricultural Research Council – Onderstepoort Veterinary Research Institute resulted in a comparable performance between FM and mCITA; however, mCITA allowed optimal growth of the fastidious *B. ovis*, which is generally inhibited on FM. Generally, mCITA seemed to be the optimum selective medium for isolation of *Brucella* spp., while FM inhibits the growth of most fungal and bacterial contaminants. Thus, veterinary laboratories can use mCITA and/or FM but should take into consideration the detection of factious *Brucella* isolated in the country or region.

## Introduction

Bovine brucellosis is a widespread zoonotic infection affecting animals and humans. It is of veterinary, public health and economic importance, as it causes substantial losses of livestock, thus affecting global trade and the livelihood of communities relying on their animals for survival (Maudlin, Eisler & Welburn 2009; Seimenis 2008). The disease is primarily caused by biovars of *Brucella abortus*, and less frequently by *Brucella suis* and *Brucella melitensis*, which are intracellular organisms and members of the genus *Brucella* (OIE 2016). The above-mentioned three species (spp.) as well as *Brucella canis, Brucella neotomae* and *Brucella ovis* are classical *Brucella* species listed in the 1980 approved lists of bacterial names (Corbel & Brinley-Morgan 1982; OIE 2016). Other members of the genus include the atypical *Brucella ceti* and *Brucella pinnipedialis* (Foster et al. 2007), *Brucella microti* (Scholz et al. 2008), *Brucella inopinata* (Scholz et al. 2010), *Brucella papionis* from baboons (*Papio* spp.) and *Brucella vulpis* from red foxes (Scholz & Vergnaud 2013; Scholz et al. 2016; Whatmore et al. 2014).

In South Africa, previous reports showed that *B. abortus biovar* (*bv.*) 1 causes 90% of infection cases in cattle, while the remaining 10% of the cases is because of *B. abortus bv*. 2 (Bishop et al. 1994; Godfroid et al. 2004). However, *B. melitensis* has been reported in slaughtered cattle in a recent study (Kolo et al. 2018).

*Brucella* control and eradication programmes should involve diagnostic assays that will address the development and transmissions of the causal organisms while taking into account local regulations and the suspected species occurring in the country (Christopher & Upadyaya 2014; Nicoletti 1984). Brucellosis has no clinical feature that allows accurate diagnosis except for abortion, which also occurs in other diseases like heartwater, salmonellosis, Rift Valley fever, leptospirosis and others (Diaz, Casanova, Ariza & Moriyón 2011; Ferreira et al. 2003).

Moreover, precise diagnosis of the disease is made difficult by several factors that include varying incubation periods, type of specimens, sensitivity and specificity of tests (especially serological assays), and multiple testing to confirm status of the herd/animals. Therefore, isolation of the organism is still regarded as the gold standard, compared with other techniques (McGiven 2003; OIE 2016), irrespective of the fact that cultures are not always sensitive, and it is time-consuming to culture and complete the phenotypic traits involved in identifying the bacteria because *Brucella* spp. are slow-growing organisms (Corbel & Brinley-Morgan 1982; Poester et al. 2010). Lack of resources also have a negative impact on diagnosis because isolation of *Brucella bv*. species can only be done in an appropriate biosafety laboratory (biosafety level 2+ and above).

Samples for the isolation of *Brucella* are mostly chosen based on the observed symptoms. They usually include stomach contents of the aborted foetuses, foetal membranes, vaginal secretions, milk, hygroma fluids, tissue samples from the udder, mammary and genital lymphs, and others. In animals, *Brucella* organisms can be obtained mostly from the infected placenta as well as from milk and lymph nodes, whereas in humans it can be isolated from blood, urine and cerebrospinal fluid (Bishop et al. 1994; Corbel 1997; Poester et al. 2010). A broad spectrum of culture media is available to grow *Brucella bv.* species and all have their own advantages and disadvantages. Biphasic media and broth are usually preferred when culturing liquid specimens, whereas solid media with 2.5% agar can be used to grow other specimens (Castaneda 1947; Poester et al. 2010). Selective media are recommended and used for the isolation of the slow-growing *Brucella* spp. because they have the capacity to inhibit the growth of most commensal and environmental bacteria (De Miguel et al. 2011; Farrell 1974; Marin et al. 1996). Colonies of *Brucella* spp. usually appear after 2–30 days of incubation, and these are transparent with smooth surfaces and intact borders (Alton et al. 1988; Da Silva Mol et al. 2012).

Veterinary diagnostic laboratories in South Africa and many other countries use Farrell’s medium (FM) to culture *Brucella* from field samples. Moreover, the Office International des Epizooties (OIE) recommends the use of both FM and modified Thayer Martin (mTM) as the nalidixic acid, and bacitracin in FM is inhibitory for *B. ovis* and some *B. melitensis and B. abortus* biovars (Marin et al. 1996). Farrell’s medium was developed for the isolation of *B. abortus* from contaminated samples (Farrell 1974), while Thayer Martin medium was developed in 1964 for the isolation of *Neisseria gonorrhoeae* and *Neisseria meningitidis* (Thayer & Martin 1964). The latter medium was later modified after the withdrawal of ristocetin, which was used to inhibit the growth of Gram-positive bacteria (Thayer & Martin 1966). It is important to use a selective media that allow the growth of the more fastidious *Brucella bv.* species like *B. abortus bv*. 2, *B. ovis* and *B. canis*. Previous studies showed that the concentration of some of the antibiotics contained in various selective media can inhibit the growth of some strains of *B. ovis, B. melitensis* and *B. abortus bv*. 2, 3 and 4 (Corbel & MacMillan 1998; Marin et al. 1996; OIE 2016). De Miguel et al. (2011) reported Agrifood Research and Technology Center of Aragon (CITA) medium, which is more sensitive than both mTM and FM for isolation of all smooth *Brucella* species while inhibiting most contaminant microorganisms. The OIE (2016) recommended the use of FM with either mTM or CITA for maximal isolation of Brucellae. The main aim of this study was to determine the medium composed of applicable antibiotic concentrations that will allow and support the growth of *Brucella* spp. causing brucellosis in South Africa while inhibiting the growth of moulds, yeast and other Gram-negative contaminants.

## Materials and methods

### Samples

Freeze-dried *B. abortus bv*. 2 strain 2534/15 and *B. ovis* strain RC48 were obtained from Agricultural Research Council-Onderstepoort Veterinary Research institute (ARC-OVR) storage collection and cultured on blood agar at 37° C in a 10% carbon dioxide incubator. The two species were opted for use in the controlled study because they are fastidious as reported in Corner and Alton (1982). The freshly grown colonies of the above-mentioned strains were harvested and suspended in phosphate-buffered saline (PBS) and used in a controlled experiment. Various samples brought in from different provinces of South Africa for routine diagnostic screening at ARC-OVR were used to further test Agrifood Research and Technology Center of Aragon (CITA) medium mCITA in conjunction with FM in collaboration with the general bacteriology section in the organisation. Both media were also used to isolate *Brucella* from milk collected from the seropositive animals during the study.

### Comparison of three different media in a controlled experiment

Farrell’s medium, mTM and CITA media were used in a controlled study to determine the optimum selective medium for isolation of *Brucella* from field samples. Gonococcus (GC) agar base and blood agar base no. 2 (Thermo Scientific™ Oxoid™, Gauteng, South Africa), foetal bovine serum (Highveld Biologicals, Johannesburg, South Africa) and the required antibiotics (Sigma-Aldrich; Johannesburg, South Africa) were used for the preparation of both mTM and CITA (Table 1), as described in a previous report by De Miguel et al. (2011), whereas FM was obtained from Selecta Media, Johannesburg, South Africa. Raw and pasteurised milk were obtained from a nearby dairy farm. The milk (9 mL) was spiked with 1 mL of bacterial suspension prepared by suspending freshly grown *B. abortus bv*. 2 strain 2534/15 and *B. ovis* strain RC48 strains in PBS and adjusted to an initial concentration of 1 × 10^5^ colony-forming units (CFU)/mL. The spiked milk was then diluted to 1:10, 1:100 and 1:1000 concentrations from which 0.1 mL/plate of each was spread and cultured on FM, mTM and CITA media in triplicate. Unspiked raw and pasteurised milk were also inoculated on all the three media as negative controls to monitor any contamination.

**TABLE 1:** Compositions and enhancements of *Brucella* selective media (Farrell’s medium, modified Thayer Martin and CITA) collated in the study.

Components	Concentrations/litre		
	FM	mTM	CITA
**Agar base**	40 g (*Brucella* medium base)	40 g (GC agar base)	40 g (blood agar base no. 2)
**Foetal bovine serum**	5%	5%	5%
**Polymyxin B sulphate**	5 mg	-	-
**Bacitracin**	25 mg	-	-
**Natamycin**	50 mg	20 mg†	20 mg†
**Nalidixic acid**	5 mg	-	-
**Amphotericin B**	-	-	4 mg
**Vancomycin**	20 mg	3 mg	20 mg
**Nystatin**	17.7 mg	17.7 mg	17.7 mg
**Colistin**	-	7.5 mg	7.5 mg
**Nitrofurantoin**	-	10 mg	10 mg

*Source:* Adapted from De Miguel, M.J., Marín, C.M., Muñoz, P.M., Dieste, L., Grilló, M.J. & Blasco, J.M., 2011, ‘Development of a selective culture medium for primary isolation of the main Brucella species’, Journal of Clinical Microbiology 49(4), 1458–1463. https://doi.org/10.1128/JCM.02301-10

mTM, modified Thayer Martin; FM, Farrell’s medium; GC, gonococcus; CITA, Agrifood Research and Technology Center of Aragon.

† , Introduction of natamycin resulting in modified mTM and mCITA.

Plates were incubated at 37 °C in a 5% – 10% carbon dioxide incubator and monitored every day for any bacterial or contaminant growth for up to 6 days, and the experiment was terminated because of high number of fungi and contaminants and thus too numerous to count (TNTC). Therefore, the composition of CITA and mTM was slightly modified by introducing natamycin to reduce the growth of contaminants and referred to as modified modified Thayer Martin (mTM) and modified CITA (mCITA). Although it was noted that the agar bases used in the media involved were different, they were not changed. That was because the main focus was to optimise the antifungals/antibacterials to reduce the growth of contaminants, which negatively affect the growth of *Brucella* spp., which are slow-growing pathogens isolated from field or clinical samples that are heavily contaminated in most cases. The controlled experiment was repeated using mCITA, mTM and FM (Table 1), as described above. The number of suspect *Brucella* CFU, contaminants CFU and contaminated plates because of fungi was recorded over 10 days of incubation at 37 °C in 5% – 10% carbon dioxide incubator.

## Isolation of Brucella from the diagnostic samples using modified Agrifood Research and Technology Center of Aragon and Farrell’s medium

The performance of mCITA medium was further validated under field condition in collaboration with ARC-OVR, which uses FM for the isolation of *Brucella* from field samples submitted for routine screening. Based on the overall performance of mTM medium in the controlled experiment, it was not considered for use in the isolation of the bacteria from field samples. Both media were simultaneously used to culture bovine tissue samples such as aborted foetuses (abomasal fluids, liver, spleen), lymph nodes, mammary glands, spleen, uterus, tonsils, as well as semen, hygromas and fluids. Samples used were submitted between January and September 2017, mostly from Gauteng and North West provinces of South Africa. All the tissue samples were homogenised using mortar and pestle method and the equal sample amounts were cultured in duplicate on mCITA and FM. Plates were incubated as described above and monitored every 2 days from days 2 to 12 following the ARC-OVR standard operating procedure (SOP). Milk from the seropositive animals sampled from two farms involved in the study was also cultured at the ARC-OVR bacteriology laboratory and biotyped at ARC-OVR.

Culture was considered positive when at least one CFU of *Brucella* was isolated. The plates were examined every 2 days for suspect *Brucella* CFU over the period of 12 days. Any visible suspected *Brucella* colonies were subcultured, typed and identified using standard microbiology procedures (OIE 2016). Briefly, *Brucella* isolates were biotyped based on their colony morphology, reaction to oxidase, urease and catalase tests, production of hydrogen sulphide, agglutination on anti-*Brucella* mono-specific sera *abortus* and *melitensis*, growth in the absence of carbon dioxide and growth in the presence of basic fuchsin and thionin dyes.

Moreover, lysis by different phages (Tb, Wb, Fi and Iz1) as well as inhibition by erythritol (1000 *µ*g) and antibiotics (Streptomycin 10 *µ*g, Penicillin G 10 units and Rifampicin 30 *µ*g) (Tbilisi; Weybridge; Izatnagar1; Berkeley2) (according to the OIE Terrestrial manual 2016) were also performed. *Brucella abortus bv*. 1 culture was used as a control in all the phenotypic tests.

## Data analysis

The average (mean) and standard deviation of CFU/mL at each dilution inoculated on mCITA, mTM and FM were determined on Microsoft Excel. Statistical comparison of the means was performed using one-way analysis of variance (ANOVA) with a Fisher’s least significant difference (LSD) post hoc test in XLSTAT (statistical software for Excel) version 2018.6.

### Ethical considerations

The experimental protocols were approved by the Animal Experiments and Ethics Committee of the University of Pretoria (V096-15 AEC Approval) and the Section 20 approval obtained from DAFF (SDAH-Epidem 15012613530_ Section 20) for the use of animals and animal products.

## Results

### Comparison of three different media (Farrell’s medium, modified Thayer Martin and Agrifood Research and Technology Center of Aragon) in a controlled experiment

**FIGURE 1:**
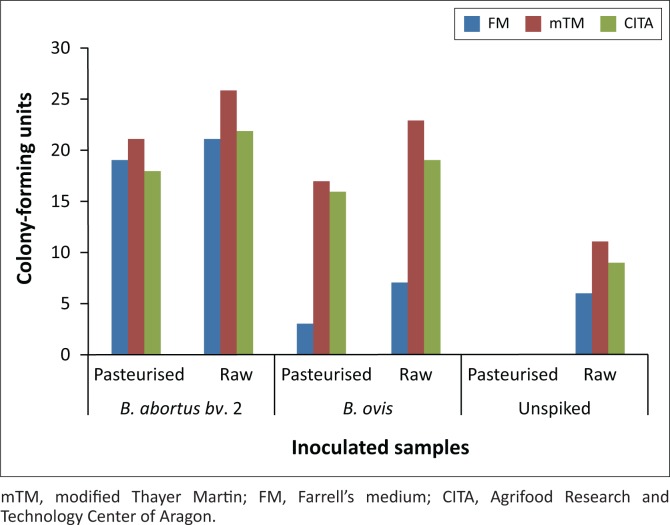
Total colony-forming unit per millilitre (colony-forming unit/mL of both *Brucella* and contaminants) count over a period of 6 days of inoculated milk samples with *Brucella abortus biovar* 2, *Brucella ovis* and unspiked. The colony-forming units are the average at day 6 of the 0.1 mL of 1:10, 1:100 and 1:1000 dilutions of pasteurised and raw milk spiked with *Brucella abortus bv*. 2 strain 2534/15 or *Brucella ovis* strain RC48 as well as unspiked pasteurised and raw milk inoculated on FM, modified Thayer Martin and CITA media after being incubated at 37 °C in 5% – 10% CO_2_. The experiment was terminated because of overgrowth of fungi on most plates inoculated with raw milk, thus making it impossible to count *Brucella*-specific bacterial colonies.

Growth was observed from day 2 on the mTM plates inoculated with raw spiked and unspiked milk, but none of the colonies were suspected to be *Brucella*. Tiny suspect *Brucella* colonies were observed from day 3 on all mTM, FM and CITA media plates inoculated with 1:10 dilution suspension. Irrespective of a high number of contaminants on mTM plates, suspect *Brucella* colonies grew on this medium at a lower rate (Figure 1; Figure 1-A1). *Brucella ovis* displayed poor growth rate on FM compared with *B. abortus bv.* 2 (Figure 1). On CITA, both strains grew at a lower rate as well; however, the number of contaminants observed was lower than those on mTM (Figure 1). The experiment was terminated on day 6 because of overgrowth of fungi on most plates inoculated with raw milk, thus making it impossible to count and differentiate *Brucella*-specific bacterial colonies.

### Comparison using Farrell’s media and the optimised modified Thayer Martin and modified Agrifood Research and Technology Center of Aragon

CITA and mTM were modified by introducing natamycin (20 mg/L) (Table 1) to enhance the performance of both media, especially decreasing the growth rate of fungal and bacterial contaminants on both media, as they make it difficult to isolate slow-growing *Brucella* colonies. Using the mCITA, FM and MTM, all the control plates inoculated with unspiked pasteurised milk did not show any growth throughout the experiment (Table 1-A1). Modified mTM medium still showed a higher number of contaminants as the initial experiment before optimisation; however, there was an improvement with mCITA, and a few contaminants were recorded as shown in Table 1-A1. The performance of FM was consistent throughout the study, and the only setback with this medium was the inhibition of *B. ovis*, as shown in previous studies as well.

In terms of *Brucella* growth, suspect colonies were visible from day 2 on the mCITA and FM as compared with day 3 with MTM, which had the highest bacterial growth rate but mostly being contaminants (Table 1-A1).

**FIGURE 2:**
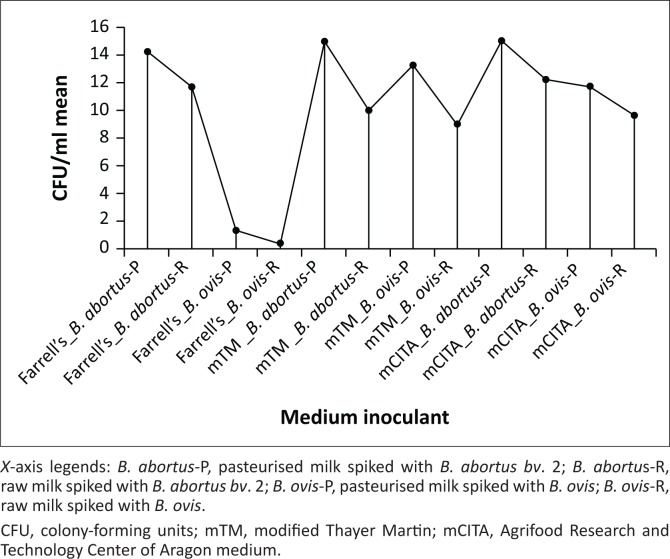
Comparison of the colony-forming unit/mL (mean) from all the plates of modified CITA, modified Thayer Martin and Farrell’s medium inoculated with pasteurised or raw milk spiked with *Brucella abortus biovar* 2 strain 2534/15 and *Brucella ovis* strain RC48; performed using one-way analysis of variance with a Fisher’s least significant difference.

The CFU mean comparison showed that the sensitivity of all the media used was comparable for the isolation of *B. abortus bv*. 2 in pasteurised and raw milk, with the mean difference ranging from 0 to 2.33 (Figure 2; Table 2-A1). Moreover, CFU mean ± standard deviation (SD) of *B. ovis* strain RC48 was generally lower in FM, irrespective of the milk type (1.3 ± 1.5 in pasteurised and 0.33 ± 0.57 in raw milk), which resulted in high mean difference when compared with mCITA (10.33) and mTM (12) (Figure 2; Table 2-A1). However, both mCITA and mTM media showed a mean difference of 0.67 and 1.67 when, respectively, using pasteurised and raw milk spiked with *B. ovis*.

### Testing modified CITA and Farrell’s medium on diagnostic samples

The mCITA was further investigated in collaboration with ARC-OVR bacteriology section using samples received for diagnostic screening. Different sample types (Table 2) were inoculated on FM and mCITA and the plates were, respectively, observed on days 2, 4, 6, 8 and 10 for any bacterial growth. Suspect, tiny *Brucella* colonies were visible from as early as day 2 on both media. Suspect *Brucella* colonies growing on mCITA did not grow when transferred or subcultured on FM. The performance rate of both media did not vary and the incubation period was similar; however, *B. ovis* grew very well on mCITA as compared with FM, as reported in previous studies. Farrell’s medium inhibited the growth of most contaminants as compared with mCITA, irrespective of the inhibition of *B. ovis*. All suspect isolates were subcultured, typed and identified as *B abortus bv*. 1, *B. abortus bv*. 2, *B. abortus* vaccine S19 and *B. ovis* (Table 2).

**TABLE 2:** List of bovine diagnostic samples, type as well as biotyping identity and the days when suspect *Brucella* colonies of each sample were initially observed on both CITA with modifications and Farrell’s medium.

Sample name	Origin	Sample type	Day when *Brucella* suspect colonies were observed	Biotyping ID‡
**SA-4408-1**	Free State	Urinary tract	-	N/A
**SA-4408-2**	Free State	Liver (aborted foetus)	4th	*Brucella abortus biovar* 1
**SA-4408-3**	Free State	Lung (aborted foetus)	-	N/A
**SA-4408-4**	Free State	Spleen (aborted foetus)	4th	*Brucella abortus biovar* 1
**SA-5423-1**	Western Cape	Suspect culture plate	2nd	*Brucella abortus biovar* 1
**SA-5423-2**	Western Cape	Suspect culture plate	2nd	*Brucella abortus biovar* 1
**SA-5423-3**	Western Cape	Suspect culture plate	2nd	*Brucella abortus biovar* 1
**SA-5513**	Gauteng	Aborted foetus (abomasal fluid)	4th	*Brucella abortus biovar* 2
**SA-5569**	Mpumalanga	Hygroma fluid	4th	*Brucella abortus biovar* 2
**SA-5706-1**	Western Cape	Suspect culture plate	2nd	*Brucella abortus biovar* 1
**SA-5706-2**	Western Cape	Suspect culture plate	2nd	*B. abortus* S19
**SA-5706-3**	Western Cape	Suspect culture plate	2nd	*Brucella abortus biovar* 1
**SA-97†**	Gauteng_F2§	Milk	4th	*Brucella abortus biovar* 1
**SA-S51†**	Gauteng_F2§	Milk	4th	*Brucella abortus biovar* 1
**SA-594†**	Gauteng_F1¶	Milk	4th	*Brucella abortus biovar* 1
**SA-1258†**	Gauteng_F1¶	Milk	4th	*Brucella abortus biovar* 1
**SA-JERSEY†**	Gauteng_F1¶	Milk	4th	*Brucella abortus biovar* 1

N/A, not applicable.

† , Milk samples collected from two farms with a history of brucellosis.

‡ , All isolates were further confirmed with abortus, melitensis, ovis & suis (AMOS), Bruceladder, multi locus variable number of tandem repeat analysis (MLVA) and real-time Polymerase chain reaction assays.

§ , Farm 2 from Bronkhorstspruit, Gauteng.

¶ , Farm 1 from Springs, Gauteng.

## Discussion

Isolation of *Brucella* bacterium is still considered the gold standard in the diagnosis of the disease because it is the only test allowing a definite diagnosis presently; however, the availability of tests that allows direct isolation of deoxyribonucleic acid (DNA) from tissue and liquid specimens is considered as a convenient alternative. In this study, we conducted a controlled experiment to determine a selective media that will allow a justifiable performance in the isolation of all *Brucella* spp. It was shown that CITA medium allows the growth of majority of *Brucella* spp. including the fastidious *B. abortus bv*. 2 and *B. ovis*, whereas the growth of the latter species was suppressed in FM. Slight modification of CITA medium (mCITA) was necessary because of the observed low inhibition rate of contaminants, especially on plates inoculated with raw milk. A higher isolation rate for *B. abortus bv*. 2 and *B. ovis* was observed with mCITA compared with FM. Amphotericin B and nystatin were used in CITA at a respective concentration of 4 and 17.7 mg/L, to control fungal infection (De Miguel et al. 2011). However, this concentration could not be increased because of the risk of toxicity that may inhibit the growth of *Brucella* species, especially *B. abortus bv*. 2 and 4 (Farrell & Robertson 1967). Thus, natamycin was introduced as an alternative supplementary fungal inhibitor because there is no reported synergy between this antifungal and the other two (amphotericin b and nystatin) used in the media, as shown in previous studies (Ghannoum & Rice 1999; te Welscher et al. 2008, Welscher 2012).

Natamycin, a polyene antifungal antibiotic produced by *Streptomyces natalensis*, is highly active against various fungi and yeasts such as *Histoplasma capsulatum, Aspergillus niger, Candida albicans* and others (Struyk et al. 1957–1958). It is used as fungi growth inhibitor because it is able to bind specifically to ergosterol, thus inhibiting the functioning of the membrane transport protein (te Welscher et al. 2008, 2012). Nystatin, filipin and amphotericin B disrupt the permeability of the membrane and the cytoplasmic features of the pathogen by interacting with the steroids available in the membrane (De Miguel et al. 2011; te Welscher et al. 2008). In this study, the inhibition of fungal growth by natamycin was shown by the performance of FM, which inhibited the growth of most fungal contaminants throughout the study in spite of the medium inhibiting the growth of *B. ovis* as well. Previous studies have shown that amphotericin B and natamycin have different modes of action even though they both belong to the macrolide polyene class of antibiotics (Ciesielski et al. 2016; Lalitha et al. 2010). Furthermore, te Welscher et al. (2008) also reported that natamycin, filipin and nystatin have different mode of action as well. Based on their findings, we suggested that it may be the reason why the use of nystatin, natamycin and amphotericin B together in one media did not show any negative effect on bacterial growth in this study. Natamycin showed very high minimum inhibiting concentration (MIC) against most bacteria as compared with fungi because the sterol-lacking membrane available in bacteria enables them to tolerate this antibiotic (De Boer 1988).

Furthermore, Ghannoum and Rice (1999) indicated that there is a significant variation between the structures of the bacteria and fungi; thus, antifungals and antibacterials target the features or functions of ergosterol and the contaminant fungal organisms, respectively.

In a report by Oostendorp (1981), it was indicated that natamycin lacks acute toxicity and the minimum LD_50_ was found to be 2.5 mg/kg – 4.5 mg/kg in animal studies. Adding natamycin to mTM and CITA made a crucial difference because most contaminants were inhibited as compared with when using the original media, even though CITA medium performed much better than mTM. In spite of the slight modification, findings in our study correspond with previous studies (De Miguel et al. 2011; De Nardi Júnior et al. 2015; Vicente et al. 2014), where the medium also displayed higher efficacy in supporting the growth of *Brucella* spp. as compared with other media used in those studies. The varying performance of CITA observed in this study and previous reports might be because of different field conditions between the countries tested and South Africa (De Miguel et al. 2011; De Nardi Júnior et al. 2015; Vicente et al. 2014), thus indicating the necessity of optimising the media in the diagnosis of brucellosis while using local samples.

The use of a selective medium that could suppress fungal, yeast and bacterial contaminant growth has been previously reported to improve isolation of *Brucella* spp. (De Miguel et al. 2011; Farrell 1974; Marin et al. 1996). Farrell’s medium is evidently the most used medium for the isolation of *Brucella* spp. however, the concentration of nalidixic acid and bacitracin in this medium inhibits the growth of other *Brucella* spp. like *B. ovis, B. abortus bv*. 2 and other biotypes of *B. melitensis* (Marin et al. 1996). Therefore, the OIE recommends the use of two media preferably FM and mTM simultaneously to overcome the setback effected by these antibiotics. This setback led to the development of various selective media ever since FM was first reported (De Miguel et al. 2011; Ewalt et al. 1983; Hornsby et al. 2000). CITA is one of the developed media (De Miguel et al. 2011), and it is well recommended for isolation of *Brucella* spp. by the OIE (2016) because of the reliable efficiency reported in several studies (De Miguel et al. 2011; Vicente et al. 2014).

The use of *B. abortus bv*. 2 and *B. ovis* strain in the controlled experiment was because of their fastidious growth and reports from previous studies, suggesting that a selective medium optimal for the growth of these species will also allow the growth of less fastidious *Brucella* spp. (Corner & Alton 1982). Moreover, isolation of *Brucella* from various sample types and different field conditions during validation of the optimised CITA indicated that the media can be used for isolation of *Brucella* from a wide range of sample types and environmental conditions. Recent reports indicated that pathogens express most of their genes based on the environmental and distinct conditions as well as the accessible nutritional sources (Wareth, Melzer & Neubauer 2017). The above-mentioned authors also suspect that pathogens can undergo some mutations to adapt to the distinct pressure in the laboratory, irrespective of their natural environment encounters, but this needs to be further investigated.

Raw milk serves as a favourable environment and source of nutrients for majority of fungal and commensal bacteria species (Delavenne et al. 2011), negatively affecting the isolation of the slower-growing *Brucella* species. As indicated in the results, the growth rate of both *B. abortus bv*. 2 and *B. ovis* in raw milk was lower than pasteurised milk in all the three media. This corresponds with a previous study by Falenski et al. (2011), which indicated that the initial concentration of 5 × 10^7^ CFU/mL of *B. abortus* 1119-3 added to raw milk dropped to 2 × 10^7^ CFU/mL within 4 days in contrast to when added to ultra high temperature (UHT)-milk, which showed an increased range of 1.5 × 10^8^ – 7.2 × 108 CFU/mL from 2 to 46 days, respectively.

In spite of the evidence that *Brucella* pathogens are difficult to grow and time-consuming because of the phenotypic processes involved in biotyping, isolation of these bacteria is still observed as the gold standard in the diagnosis of brucellosis (OIE 2016). In addition, it is not certain whether *Brucella* growth will be obtained during culturing in spite of its specificity and even if the sample is from a known positive animal with a chronic infection that does not shed the bacteria (Capparelli et al. 2009); thus, in this study, we used different sample types from one animal to improve isolation as well to avoid missing any possible co-infection. Alton et al. (1988) indicated that a standard basal media could also be used to isolate the majority of *Brucella* spp. nonetheless, this is not practical for the initial isolation of Brucellae from field clinical samples. This may be because of various factors like sample collection, transportation, storage, available expertise, availability of viable bacteria in samples and so on (Corner & Alton 1982). Based on the sample type as well as the distance between the collection site and the laboratory, it is of utmost importance to store and transport the samples send for routine screening/diagnosis properly to avoid contamination or sample spoilage (Alton et al. 1988).

*Brucella* isolation requires expertise and it is laborious; hence, it is necessary to further investigate any suspect colony observed on each plate with staining methods to avoid misdiagnosing the bacteria (OIE 2016). Staining methods are inexpensive, rapid and user-friendly tests used for the presumptive identification of *Brucella* suspect colonies. However, other Gram-negative bacteria like *Coxiella burnetii* and *Chlamydia psittaci* can be morphologically equivalent to *Brucella* spp. on the most commonly used modified Ziehl-Neelsen staining (Poester et al. 2010; Stamp et al. 1950). Tilak et al. (2016) also reported misidentification of five *Brevundimonas diminuta* isolates as *Brucella* spp. using Stamp’s modified Ziehl-Neelsen staining because they also stained pink against the blue background.

Typically, *Brucella* can be easily detected in endemic areas as compared with areas with low infection rate where some of the cases might be overlooked (Jimenez de Bagues et al. 1991). Therefore, the use of an efficient and reliable medium can overcome the disadvantages encountered with the various sample types and their conditions. In this study, CITA with slight modification has proven to be more sensitive and regarded as the medium of choice in the isolation of all *Brucella* spp., as indicated in previous studies.

Even though the optimisation and modification done on the formulation of CITA medium were not major, the efficacy of this medium was improved when culturing field and diagnostic samples in this study. Thus, the use of this medium, or in conjunction with FM, may improve the overall isolation of these intracellular, fastidious and slow-growing pathogens.

## Appendix 1

**TABLE 1-A1:** Total colony-forming unit per plate (CFU/plate) for 1:10 dilution of all three media over a period of 12 days.

Medium	Total CFU	*Brucella* suspect CFU	Day contaminant colonies observed	Day *Brucella* suspect colonies observed
**B. *abortus* bv 2**				
**Pasteurized milk**				
**FM**	29	22	4	2
**MTM**	32	26	3	2
**CITA**	31	24	4	2
**Raw milk**				
**FM**	33	19	3	3
**MTM**	39	17	2	3
**CITA**	35	18	3	2
***B. ovis***				
**Pasteurized milk**				
**FM**	7	4	4	4
**MTM**	30	21	3	2
**CITA**	26	19	4	2
**Raw milk**				
**FM**	14	2	4	5
**MTM**	39	15	2	3
**CITA**	29	16	3	3
**Unspiked**				
**Pasteurized milk**				
**FM**	0	0	NONE	NONE
**MTM**	0	0	NONE	NONE
**CITA**	0	0	NONE	NONE
**Raw milk**				
**FM**	9	0	3	NONE
**MTM**	14	0	2	NONE
**CITA**	9	0	3	NONE

Note: Pasteurised and raw milk spiked with 1 x 10^5^ CFU/ml of *B. abortus* bv 2 strain 2534/15 and *B. ovis* strain RC48 as well as unspiked pasteurised and raw milk were inoculated on Farrell’s (FM), modified modified Thayer Martin (MTM) and modified CITA media, then incubated at 37 °C in 5 % – 10% CO_2_. Total CFU, *Brucella* CFU and days when suspect *Brucella*/contaminant colonies were initially observed on FM, MTM and mCITA media are indicated.

MTM, Thayer Martin; CITA, Agrifood Research and Technology Center of Aragon; CFU, colony-forming units; FM, Farrell’s medium; mCITA, Agrifood Research and Technology Center of Aragon medium; *B. abortus, Brucella abortus; B ovis, Brucella ovis*.
